# Adolescent pregnancies and adherence to puerperal consultation*

**DOI:** 10.1590/1518-8345.6269.3702

**Published:** 2022-10-03

**Authors:** Ingrid Rosane Pinto, Jéssica Aparecida da Silva, Patrícia Casale Parra, Monika Wernet, Luciana Mara Monti Fonseca, Mariana Torreglosa Ruiz

**Affiliations:** 1 Universidade Federal do Triângulo Mineiro, Uberaba, MG, Brasil.; 2 Universidade Federal de São Carlos, São Carlos, SP, Brasil.; 3 Universidade Federal de São Carlos, Departamento de Enfermagem, São Carlos, SP, Brasil.; 4 Universidade de São Paulo, Escola de Enfermagem de Ribeirão Preto, Centro Colaborador da OPAS/OMS para o Desenvolvimento da Pesquisa em Enfermagem, Ribeirão Preto, SP, Brasil.; 5 Universidade Federal do Triângulo Mineiro, Departamento de Enfermagem na Assistência Hospitalar, Uberaba, MG, Brasil.

**Keywords:** Adolescent, Pregnancy in Adolescence, Postpartum Period, Prevalence, Disease Prevention, Patient Compliance

## Abstract

**Objective:**

to determine the profile of pregnancies and prevalence of adherence to puerperal consultation among adolescent puerperal women compared to non-adolescent puerperal women served in an outpatient clinic of a teaching hospital in the rural area of Minas Gerais.

**Method:**

cross-sectional study nested in a cohort of puerperal women; non-probabilistic sample, by convenience; adolescent pregnancy - dependent variable; sociodemographic, clinical and obstetric - independent variables. It employed its own instrument, tested by means of a pilot test. Prevalence ratios and confidence intervals were calculated; chi-square and Fisher’s exact tests were applied, considering a significance level of 5%, and Poisson regression with robust variance.

**Results:**

we interviewed 121 puerperal women, of which 18.2% (22) were adolescents, and observed among them low educational level (p<0.001); fewer pregnancies with pathologies (p=0.016); predominance of primiparous women (p<0.001), and higher rates of normal delivery (p=0.032). The prevalence of adherence to puerperal consultation was 34.7% and 31.8% for adolescents. There were no differences regarding adherence and age of puerperal women.

**Conclusion:**

adolescents did not present negative obstetric and neonatal outcomes, although a lower educational level was observed. Association was found between early age and absence of diseases during pregnancy and higher rates of normal vaginal deliveries. Adherence to puerperal return visit was slightly lower, but without statistical significance.

## Highlights

(1) Adolescent puerperal women had low educational level.

(2) Association between early age and absence of diseases during pregnancy.

(3) There were higher rates of normal vaginal deliveries among adolescents.

(4) Adolescents did not present negative obstetric and neonatal outcomes.

(5) There were no differences regarding adherence and age of puerperal women.

## Introduction

Adolescents represent about 40% of the Brazilian population^1^. The World Health Organization (WHO) considers that adolescence is the phase of life between childhood and adulthood, involving the age group from 10 to 19 years^1^. During adolescence, there are rapid and progressive physical, cognitive and psychosocial changes that tend to affect the way of feeling, thinking, making decisions and interacting with the world. This is a significant period for the constitution of subjectivities, choice of behaviors and establishment of identity^1^.

Among the numerous issues related to adolescence are sexuality, gender, and sexual and reproductive behavior. A Brazilian study showed that 27.5% of adolescents had already initiated sexual activity and, of these, 66% used condoms^2^. A study carried out with 499 adolescents aged between 12 and 17 years showed, in turn, that 47.3% of them had already initiated sexual activities; the mean age of first sexual intercourse was 14.1 years, with a tendency of early sexual initiation in males, with one third of the first sexual intercourses having been unprotected (33.9%)^3^.

Early and unprotected sexual activity increases the chances of adolescent pregnancy. Data from the WHO indicate that every year more than 21 million girls aged between 15 and 19 years become pregnant worldwide and, of these, more than 10 million pregnancies are not planned^4^.

In the Brazilian territory, records from the Department of Informatics of the Unified Health System (DATASUS) indicate more than 29 million births in the period from 2010 to 2019. Of these, twelve mothers were aged under 10 years; 252,000 (0.9%) were aged between 10 and 14 years, and about 5 million (17%) were aged between 15 and 19 years^5^. During this period, adolescent pregnancies represented about 18% of all births in the Brazilian territory and showed a stable trend^5^.

Adolescent mothers are at a higher risk of developing preeclampsia, puerperal endometritis and other infections; while their newborn children are at a higher risk of low birth weight and premature birth^6^. Moreover, pregnant adolescents who are not in a stable relationship may experience stigma, rejection and violence of all kinds from their sexual partners, parents and even their peers^7^ ^-^ ^8^.

Pregnancies and births in adolescence tend to increase school dropout rates^9^, an aspect that contributes to the perpetuation of the cycle of low educational level associated with low wages, scarce employment and training opportunities, thereby increasing social inequalities^10^.

Considering its impacts, the WHO and the Brazilian Ministry of Health (MS) classify adolescent pregnancy as high-risk pregnancy due to the possibility of repercussions on the health and quality of life of the mother and newborn. In this study, the objective is to evaluate the outcomes in the puerperium of women who were pregnant in adolescence. The puerperium comprises the involutive process after childbirth; with indeterminate duration, it includes hormonal and immunological changes for women^11^, in addition to being a period of intense physical and emotional vulnerability.

In order to avoid and trace complications in this period, the Brazilian Ministry of Health recommends, before hospital discharge, referral of the woman to the unit where she received prenatal care, having with her a complete report on birth and immediate and mediate postpartum evolution, and at least one consultation between 7 and 42 days after birth^12^ ^-^ ^13^. In addition, at least one home visit is recommended in the first week after discharge; however, if the newborn has been classified as at risk, it should occur in the first three days^12^ ^-^ ^13^.

Although only one puerperal consultation is recommended in the Brazilian territory, the adherence rate ranges from 16.8 to 58%^14^ ^-^ ^15^. This index is much lower than what would be desirable when compared to data from other countries such as the United Kingdom, where adherence to puerperal consultation has an index of 91%^15^.

The study is warranted, considering the magnitude of adolescent pregnancy in Brazil, the scarcity of studies on the subject of puerperium in adolescence, and the possible impacts on maternal and neonatal outcomes. The objective was to determine the profile of pregnancies and prevalence of adherence to puerperal consultation among adolescent puerperal women compared to non-adolescent puerperal women served in an outpatient clinic of a teaching hospital in the rural area of Minas Gerais.

## Method

This is a cross-sectional study nested in a cohort of puerperal women, which compared the outcomes of adolescent puerperal women with the outcomes of non-adolescent puerperal women served in an outpatient prenatal and puerperium service of a teaching hospital in the rural area of Minas Gerais. The institution provides prenatal care for pregnant women at usual risk in the health district where it is located (about 150,000 inhabitants), and is a reference for high-risk pregnancies for 27 municipalities in the Southern Triangle of Minas Gerais. Consistently with the recommendations in Brazil, puerperal women are referred to the unit where she received her prenatal care, for puerperal follow-up^13^. According to institutional data, in the data collection period - August to December 2019 - there were 573 deliveries.

The survey for potential participants and data collection were carried out by the first author of this study in the rooming-in ward of the mentioned hospital and, later, only to check attendance, in the records of the outpatient clinic.

The inclusion criteria adopted were: being a puerperal woman receiving care in the rooming-in wards of the mentioned hospital, with expected hospital discharge; being hemodynamically stable, conscious, and not disoriented; having a scheduled puerperal return visit. The following non-inclusion criteria were adopted: puerperal women whose return visit was scheduled to take place in Basic Health Units (BHU) or Family Health Units (FHUs). Thus, we screened puerperal women who would be followed up by the outpatient clinic of the hospital listed in the study.

The identification of the study criteria was performed with the nurse responsible for the rooming-in ward. Respecting the inclusion and exclusion criteria, there were no exclusions or losses during collection.

The sampling was by convenience, non-probabilistic, during the period established for data collection. We interviewed a total of 121 puerperal women, corresponding to 21% of all puerperal women served in the rooming-in wards in the period, and 100% of those who met criteria for scheduling institutional return visit.

### Data collection

Data collection was carried out prospectively at two different times. After being instructed and providing consent to participate in the study, all puerperal women were interviewed and data were supplemented with medical records. The approach, medical record data collection, and interviews were conducted by two researchers trained and calibrated by the main researcher.

The data were collected by using a specific instrument, tested through a pilot study, which did not demonstrate the need for adaptations. We obtained sociodemographic data (age; self-declared skin color/race; if they cohabited with a partner; educational level; if they had a paid activity and what activity they had), clinical data (habits such as smoking, alcohol consumption and use of illegal drugs; chronic and/or gestational diseases), and obstetric data (number of pregnancies; births; abortions; gestational age in weeks considered at the time of delivery; type of delivery; newborn birth weight; if there were postpartum complications), by means of an interview recorded in the instrument. We consulted the medical records for specific information about childbirth, neonate, indication for return visit at the institution, and incomplete information provided by the participant.

Subsequently, by referring to the list of visits scheduled for the date in the hospital’s computerized system (electronic medical records), the researchers independently verified the presence or absence in the visit scheduled for the puerperal women included in the study sample, and the answers were validated after being inputted in the database.

### Statistical analysis

The study’s dependent variable was adolescent pregnancy, according to the definition of the WHO, in the age group between 10 and 19 years. Sociodemographic, clinical and obstetric data were investigated.

After collection, the data were coded, stored in an Excel spreadsheet, with double entry technique and subsequent validation. The database was validated and imported into the Statistical Package for the Social Sciences (version 23). Initially, we conducted descriptive analyses (frequency, mean, standard deviation, minimum and maximum) of the variables and the results were presented in tables; chi-square and Fisher’s exact tests were applied considering a significance level of 5%; we estimated prevalence ratios and corresponding 95% confidence intervals.

Poisson regression with robust variance was applied in multiple analysis, being indicated for the analysis of counting data and to minimize the effects of the overestimation of the prevalence ratio that occur when the outcome is common or very frequent in the sample^16^. We used the model in which the independent variables were entered in blocks in the following order: sociodemographic, clinical and obstetric data. We included, in the model, variables with p<0.20 in the univariate analyses. The variables in the model were selected by the backward stepwise method. By this method, all variables with a value equal to 0.20 are considered in the univariate analysis for the Wald statistics in the maintenance of the variables during the analysis adjusted level by level, in order to control potential confounding factors^17^ and identify real association factors.

### Ethical aspects

After the nurse responsible for the rooming-in ward indicated a woman as a potential participant, the researcher went to the ward, invited her for the study and, if she showed interest, read together with her the Consent Form or Free and Informed Consent Form with space for clarification of doubts. In cases where the puerperal woman was aged under 18 years, her legal guardian was previously contacted, the study was presented and their consent was registered through the Informed Consent Form.

The study was approved by the Research Ethics Committee, opinion number 2.148.698, of June 30, 2017, and its entire development was based on and guided by the Regulatory Guidelines and Standards for Research involving human beings contained in Resolution 466/12/CNS/MS.

## Results

When characterizing the 121 puerperal women who participated in the study, the mean age found was 25.5 ± 6.7, ranging from 14 to 43 years, and of these, 22 (18.2%) were adolescents.

Among the adolescent pregnant women interviewed, 40.9% (09) declared themselves white; 63.6% (14) were married; only 9.1% (02) had complete secondary education, and 50% (11) were students. Regarding habits, 18.2% (04) were smokers and smoked about one pack per day; 9.1% (02) declared themselves social drinkers, and 9.1% (02) reported daily use of marijuana. Of the respondents, eight (36.4%) had some pathology, with more frequent reports of hypothyroidism (9.1%) and syphilis (9.1%).

Most adolescents (81.8% - 18) were primiparous mothers, although 13.6% (03) were pregnant for the second time, and one (4.5%) was in the third pregnancy; 13.6% (03) had previous abortion experience; all (22) had prenatal care with an adequate number of consultations (six or more consultations), and 77.3% (17) of pregnancies had normal delivery as the outcome. Most newborns (90.9% - 20) were born at term and none were born weighing less than 2500 grams. Three adolescents (13.6%) had postpartum complications: one case of placental acreticism, one case of uterine atony, and one case of postpartum hemorrhage.

The prevalence of adherence to the general puerperal consultation was 34.7%, and the index among adolescents was 31.8% (07) of return to service. There were no statistically significant differences regarding adherence and age of the puerperal women. Only two adolescents (9.1%) reported contraception in the puerperium: one had intrauterine device (IUD) inserted postpartum, and one was prescribed injectable contraceptive.

Table 1 presents comparative data of the sample characterization in relation to adolescent pregnant women and non-adolescent pregnant women interviewed. The data indicated a low level of education among adolescents; a lower number of pregnancies with pathologies; a predominance of primiparous women; and higher rates of normal vaginal delivery as the outcome.

**Table 1:** Sociodemographic, clinical and obstetric characterization of pregnancies among adolescents and non-adolescents. Uberaba, MG, 2019

Variable	Adolescents		Non-adolescents		RR	95% CI	p*
	n	%	n	%			
White skin color/race	9	7.4	47	38.8	0.862	0.501 - 1.484	0.641
White skin color/race	13	10.7	52	42.9			
Cohabiting with a partner	14	11.6	56	46.3	1.125	0.785 - 1.612	0.637
Not cohabiting with a partner	8	6.6	43	35.5			
Educational level lower than secondary education	20	16.5	47	38.8	1.876	1.470 - 2.395	<0.001
Educational level higher than secondary educational	2	1.6	50	41.3			
Smoker	4	3.3	18	15.0	1.273	0.463 -3.496	0.741
Non-smoker	14	11.7	84	70.0			
Alcoholic	2	1.6	20	16.5	0.692	0.168 - 2.850	1.000
Non-alcoholic	13	10.7	86	71.1			
Use of illegal drugs	2	1.6	1	0.8	9.000	0.854 - 94.899	0.085
Non-use of illegal drugs	20	16.5	98	80.9			
With pathologies	8	6.6	65	53.7	0.554	0.313 - 0.980	0.016
No pathologies	14	11.7	34	28.1			
Primiparous	18	14.9	24	19.8	0.240	0.098 - 0.586	<0.001
Multiparous	4	3.3	75	62.0			
Normal delivery	17	14.0	50	41.3	1.530	1.135 - 2.063	0.032
Cesarean section	5	4.1	49	40.5			
Preterm newborn	2	1.6	10	8.3	0.900	0.212 - 3.822	1.000
Full-term newborn	20	16.5	89	73.6			
Low birth weight	0	-	5	4.1	1.053	1.006 - 1.102	0.583
Appropriate weight	22	18.2	94	77.7			
Postpartum complications	3	2.5	5	4.1	2.700	0.697 - 10.465	0.158
Without complications	19	15.7	94	77.7			
Attended puerperal return visit	7	5.8	35	28.9	0.900	0.462 - 1.754	0.810
Did not attend return visit	15	12.4	64	52.9			

*95% CI; Significance level = 5%; Values with statistical significance are highlighted in bold

Table 2 presents the Poisson Robust Regression Model to explain adherence to puerperal consultation associated with sociodemographic, clinical and obstetric variables, including the relation with adolescent pregnancy. The following variables were significant: use of illegal drugs, which was associated with non-adherence, and primiparity, which explained adherence.

**Table 2:** Poisson regression model to explain adherence to puerperal consultation associated with sociodemographic, clinical and obstetric variables. Uberaba, MG, 2019

Variable	Coefficient ^*^	(95%CI)		p
Adolescence	0.002	(-0.146)	(-0.151)	0.974
Educational level	0.074	(-0.041)	(0.189)	0.207
Use of illegal drugs	0.579	(0.012)	(0.249)	0.031
With pathology(ies)	-0.101	(-0.207)	(0.004)	0.060
Primiparity	-0.131	(-0.262)	(-0.01)	0.049
Cesarean section	0.045	(-0.065)	(-0.154)	0.426
Postpartum complications	0.093	(-0.063)	(-0.250)	0.243

^*^Poisson’s Robust Regression Model, backward stepwise method, adopted p≤0.20

Thus, adolescent pregnancy did not justify adherence or non-adherence to puerperal consultation.

## Discussion

The puerperal return visit is a strategic opportunity to prevent, detect and treat changes that can be lethal and/or compromising to the health of women and, consequently, their newborns.

The women interviewed were in the age group considered as *fertile age*, as well as puerperal women who participated in other national and international studies^18^ ^-^ ^24^. However, the mean age (25 years) was lower than that found in studies conducted in the southern region of Brazil^18^ and in Poland^24^. Despite the lower age than that found in other studies, no associations were found between adherence and maternal age.

However, the adolescents constituted 18.5% of the sample of puerperal women interviewed, similarly to nationwide data^5^, which indicate rates of 18% of pregnancies in adolescence. But the study showed a higher rate than the data found in the Nascer no Brasil survey, conducted in 2011 and 2012, which showed a percentage of 11%^25^. There is evidence of a trend of increasing pregnancies among adolescents, an aspect that refers to the consideration of the existence and outreach of Primary Care actions aimed at adolescent health and sexual and reproductive planning^26^, in addition to the partnership between health and school.

The study indicated low educational level among pregnant adolescents, similarly to data found in the Nascer no Brasil survey^25^ ^,^ ^27^. A qualitative study pointed out that 75% of adolescents interrupt schooling due to an ongoing pregnancy^28^. Low educational level is a determinant to be considered by professionals. A study^29^ conducted in the United States related low educational level of puerperal women with feeling discriminated against during the hospitalization period and with low rates of adherence to postpartum return visits. The puerperal women reported having difficulties in understanding guidelines, not perceiving efforts of the professionals to adapt the language, pointing out that this contributed to their decision not to return to the service^29^. Although no association was found between education and adherence to the consultation in this study, others indicated lower adherence among women with low educational level^18^ ^,^ ^29^ ^-^ ^30^.

Regarding habits, 18.2% of the adolescents were smokers, 9.1% were alcoholics, and 9.1% reported using illegal drugs (marijuana). It is noted that the use of illegal drugs was also a factor associated with non-adherence. A study conducted in the state of Rio Grande do Sul with puerperal women showed higher rates of smoking (53.8%) and alcohol consumption (63.3%), but a lower percentage of illegal drug use (marijuana) (3.8%)^31^. Data from the Nascer no Brasil^27^ survey showed a lower rate of smoking among adolescents (8.6%) and higher alcohol consumption (11.3%) when compared to data from the present study. Other studies are required to provide further evidence about the consumption of psychoactive substances by adolescents and during pregnancy and puerperium, and about the conduct of professionals when dealing with such situations. The use of illegal drugs was associated, in this study, with the statistical significance of non-adherence in the puerperium.

The primiparity index in the sample was 34.7% and 81.8% among adolescents, results similar to those of the Nascer no Brasil study^25^ ^-^ ^27^. It is noted that an association was found between primiparity and higher adherence to puerperal return visits. Puerperal follow-up of primiparous women is strategic for the health of women and children, especially when considering home visits^32^. In the puerperal consultation, there are different chances to detect complications such as infections and other complications, and to obtain information about the health of the woman and her family, to provide guidance on family planning and to clarify doubts related to childcare and breastfeeding^33^.

All adolescent puerperal women received prenatal care in an appropriate manner, unlike the data indicated in the Nascer no Brasil studies, which showed adherence of less than 60%^25^ ^,^ ^27^.

Among the adolescents, 36.4% had pathology, and there was a greater association between the occurrence of pathologies and advancing maternal age. We found no cases of preeclampsia and/or infectious processes, which are outcomes associated with adolescent pregnancies in an international multicenter study^6^. In contrast, 9.1% of the adolescents reported a case of syphilis during pregnancy. The disease was also reported in a nationwide study on adolescent pregnancies with a frequency of 0.9%^27^. Syphilis is a public health issue and directly impacts the quality of prenatal care and also the support of health care related to sexual and reproductive health issues.

Regarding childbirth, normal deliveries were prevalent in the sample of this study (77.3%), with a rate higher than those found in nationwide studies - ranging from 62.4 to 65.7% of deliveries^25^ ^,^ ^27^. Although no association was found between the type of delivery and puerperal return visits, a qualitative study pointed out that women who had a cesarean section attributed greater importance to puerperal return and home visits due to the professional evaluation of the surgical wound^34^.

It should be noted that although the international literature reports association between adolescent pregnancies, premature births, and low birth weight^6^, such association was not found in the present study, with a prematurity rate of 9.1% and absence of low birth weight neonates among the adolescents studied.

The results of this study indicated a low rate of adherence to the puerperal consultation (34.7%) in the total sample and lower rates among adolescent puerperal women (31.8%), although without statistical association with age. Adherence was lower when compared to rates found in studies conducted in Mato Grosso do Sul (43.1%)^19^ and in the municipality of Botucatu - where the adherence rate was 46.9% in the BHUs and 69.7% in the FHUs^35^ -, in the state of Paraná (51.1%)^36^ and in the southern region of Brazil (75.2%)^18^. However, the index found is consistent with the rate indicated in a literature review study that obtained adherence rates ranging from 16.8% to 58%^14^ ^-^ ^15^. This index is much lower than what would be desirable when compared to data from the United Kingdom, where adherence to puerperal consultation has an index of 91%^15^. It should be mentioned that puerperal care is still the most critical point, considered the worst indicator of care for the pregnancy-puerperium cycle^37^. It is necessary to rethink health care in the puerperium, seeking reasons for adherence and non-adherence to puerperal follow-up.

Absent or insufficient puerperal follow-up may result in unnecessary demands for urgency and emergency services, which are geared towards providing service in cases of acute problems of high severity that require fast and immediate care, especially when there is risk of imminent death^38^.

The literature points out critical points of puerperal care: postpartum care still has as main focus the care of newborns^35^; adherence to consultation related to complications (curative rather than preventive); need for greater visibility of the woman; and improved professional training in puerperal care^32^.

Rethinking puerperal care implies tracing facilitators and barriers to adherence. A North-American study found, as factors for non-adherence, the difficulty as to transportation, the care required by other children, and the lack of professional engagement in the active search for absentees^39^. In addition to these factors, the authors found that fragmented consultations and care for the mother and the newborn make adherence difficult^39^, since the puerperal woman often prioritizes the care of the child to the detriment of self-care.

Furthermore, even recommending a single consultation, it appears that only this time is insufficient to address the particularities of the situation and provide advice on the different topics that are covered in the puerperal care, such as postpartum depression, breastfeeding, reproductive planning, healthy eating, exercise practice, etc. In order to enhance the puerperal consultation, there are indications that the care protocol could provide an informative, meaningful and quality consultation^40^. However, the professional is responsible for intervening in the uniqueness of the experience and need of each woman, child and family so the meaning and significance of the puerperal consultation is accomplished. Still, when considering that adolescence itself already poses issues, one can infer the complexity and comprehensiveness that a postpartum consultation can constitute, questioning the sufficiency of strictly following protocols.

The experience of puerperium in adolescence can awaken a sense of early maturity due to motherhood. A study pointed out that adolescents receive instrumental support from their parents, that is, help in their routine with the baby, not recognizing health professionals as support^41^. This same study found that the lack of support resulted in non-monitoring of health, school dropout, social isolation and troubled relationships with partner and/or family^41^. In contrast, home visits (HVs) are pointed out as an alternative to bond with adolescents because it is conducive to understanding of the life context and individualities of the adolescent woman^15^ ^,^ ^41^. When considering the feeling of abandonment and neglect reported by adolescent puerperal women^28^, it can be inferred that the active search and employment of home visits foster the understanding and intervention in the singularity of the situation, in addition to being a strategy for adherence and bonding with the team.

It is noted that, despite low coverage of puerperal return visits, puerperal women who are referred to the unit of origin, where they received prenatal care, are more likely to return. Thus, the quality of prenatal care is shown to enhance the minimization of socioeconomic effects and adversities in maternal and child health indicators^35^. These data may be reflected on the results of the present study, which indicated the absence of unfavorable outcomes and adherence of all pregnant adolescents to prenatal care.

Adolescent pregnancies in the Brazilian territory are classified as at high risk^12^, and, in these cases, obstetric nurses can work fully during prenatal and postnatal care^42^ ^-^ ^43^. However, their role is often restricted to the initial care and specific actions of health education^42^. It is worth mentioning that even in case the adolescent is referred to the high-risk outpatient clinic, Primary Health Care should be the coordinator of care and remain in a collaborative partnership in the monitoring of this adolescent^13^.

In addition, nurses can work with families in order to deal with the impact of motherhood at this stage of life^44^, which may have repercussions on mental health, since 26% to 50% of pregnant adolescents have postpartum depression^45^.

It is emphasized that the autonomy of nurses in providing care in the pregnancy-puerperium cycle should be based on technical and scientific training^44^; however, care in adolescent pregnancy and puerperium combines particular knowledge and skills stemming from the experience of adolescence, when finalist academics show technical-scientific and psychological unpreparedness to work with adolescents^46^. Adolescence still represents a challenge for the profession, since it consists in a group with high social vulnerability and requires qualified and specific care^47^. Thus, the subject of adolescence and pregnancies in this phase need to be expanded in health training.

Providing care to pregnant adolescents and puerperal adolescent women means providing comprehensive care sensitive to the specificities of the age group and the transition related to motherhood, with actions aimed at empowerment, self-care and reproductive planning, respecting the ethical and legal aspects of care and the rights of adolescents. The adolescent mother needs to be better informed and to be heard and respected as to her rights, without any kind of restriction for the simple fact of being an adolescent^25^.

The authors understand that a limitation of the study refers to external validity, since the data cannot be generalized to other realities. It should be noted that, based on the results found, further studies on the subject can be carried out, which can be supported by means of hypothesis tests or that use different designs.

## Conclusion

Adolescent pregnancies in the study sample did not indicate negative obstetric and neonatal outcomes, although a lower level of education was observed in this population. Despite the limitations of the study regarding its generalization, these data may reflect the importance and influence of prenatal adherence on favorable outcomes, since all of them attended the recommended consultations. It was found that early age was associated with absence of diseases during pregnancy and higher rates of normal vaginal deliveries. The prevalence of adherence to puerperal return visit was slightly lower than that of non-adolescent women, but this difference was not statistically significant.

The low adherence reinforces the importance of puerperal consultation as a tool for prevention and promotion of maternal-child and adolescent health. The use of marijuana was associated with low adherence to puerperal consultation.

The results suggest particularities related to the puerperal care of adolescent mothers, with urgency as to rethinking the framework of puerperal care and the training of health professionals for the particularities of this population.

The discussions conducted can be considered both in the institution where the study was conducted and in others, given that, in comparison with other studies, there is a need to increase adherence in different contexts and institutions.
